# The Essentiality Status of Mouse Duplicate Gene Pairs Correlates with Developmental Co-Expression Patterns

**DOI:** 10.1038/s41598-019-39894-9

**Published:** 2019-03-01

**Authors:** Mitra Kabir, Stephanie Wenlock, Andrew J. Doig, Kathryn E. Hentges

**Affiliations:** 1 Division of Evolution and Genomic Sciences, Faculty of Biology, Medicine and Health, Manchester Academic Health Science Centre, University of Manchester, Oxford Road, Manchester, M13 9PT UK; 2 Present Address: Department of Pathology, Cambridge Genomic Services, University of Cambridge, Cambridge, CB2 1QP UK; 3 Manchester Institute of Biotechnology and School of Biological Sciences, Faculty of Biology, Medicine and Health, Manchester Academic Health Science Centre, University of Manchester, 131 Princess Street, Manchester, M1 7DN UK

## Abstract

During the evolution of multicellular eukaryotes, gene duplication occurs frequently to generate new genes and/or functions. A duplicated gene may have a similar function to its ancestral gene. Therefore, it may be expected that duplicated genes are less likely to be critical for the survival of an organism, since there are multiple copies of the gene rendering each individual copy redundant. In this study, we explored the developmental expression patterns of duplicate gene pairs and the relationship between development co-expression and phenotypes resulting from the knockout of duplicate genes in the mouse. We define genes that generate lethal phenotypes in single gene knockout experiments as essential genes. We found that duplicate gene pairs comprised of two essential genes tend to be expressed at different stages of development, compared to duplicate gene pairs with at least one non-essential member, showing that the timing of developmental expression affects the ability of one paralogue to compensate for the loss of the other. Gene essentiality, developmental expression and gene duplication are thus closely linked.

## Introduction

Gene duplication is a key evolutionary event in multicellular eukaryotes^1^. It can generate new genes (paralogues), retaining sequence similarity to the ancestral gene, but performing new biological functions^2,3^. Duplication events can be small scale, involving regions containing a single gene, or encompass the entire genome. Evolutionary pressures can cause duplicated genes that are no longer useful to the organism to acquire loss-of-function mutations and become non-functional pseudogenes^4^. For a gene to be retained in the genome following duplication, it has been proposed that the gene must undergo neofunctionalisation (acquiring a new function not present in the ancestral gene) or subfunctionalisation (whereby members of a duplicated gene family each only retain a subset of the original gene functions)^5–7^. The preservation of duplicate genes may also be driven through functional conservation^2,8^ (retaining the function of the ancestral gene throughout the evolution) and neosubfunctionalisation^9,10^ (whereby a duplicated gene undergoes subfunctionalisation, followed by the acquisition of further new functions not present in the ancestral gene). Subfunctionalisation can arise from partitioning of the expression pattern of duplicated genes, such that individual duplicate genes are expressed within a subset of the temporal and morphological expression domains of the ancestral gene^11–15^. A study of duplicated genes in the human genome identified subfunctionalisation of gene expression as a rare event, which occurs after the duplicated genes have been segregated to different chromosomal locations and acquire divergence in their expression patterns^16^.

Given that selective pressures will influence gene retention within a genome after a duplication event, it may be assumed that duplicates retained as active genes each perform functions that are required within the organism. Prior studies reported that the functional loss of deleting a duplicate gene could be compensated by the existence of its close paralogue in the same genome if the paralogue retains overlapping functions and expression patterns^17–19^, although this phenomenon may be rare. Moreover, genome-wide gene knockdown or knockout experiments in *Caenorhabditis elegans*^20,21^ and *Saccharomyces cerevisiae*^17^ showed that duplicate genes are considerably less likely to be essential than singletons (single-copy genes). However, studies of mouse knockout phenotypes reported that the proportion of essential genes between singletons and duplicates is similar; therefore, mouse duplicate genes are just as essential as singletons^22,23^. Duplicate gene essentiality was attributed to connectivity in protein-protein interaction networks (PINs), based on the finding that mouse duplicate genes are highly connected within PINs, whereas duplicated yeast genes, which are less likely to be essential, are not highly interconnected within PINs^22^. The over-representation of mouse developmental genes in the dataset of genes that have been knocked out has also been proposed as an explanation for the high frequency of essential duplicate genes in mouse knockout experiments, since developmental genes would be more likely to generate a lethal knockout phenotype^24^. Further studies have reported that recently duplicated genes are under-represented in the mouse knockout dataset, but after correction for this bias singletons were more likely to be essential than duplicates^25,26^, and within a given evolutionary age group, singletons are more likely to be essential^27^.

Genes expressed during early mouse development were more likely to revert to a single copy following whole genome duplication events^28^. Highly expressed developmental genes were more likely to be essential, suggesting a positive association between singleton developmental genes and essentiality^28^. However, to our knowledge no previous studies have examined the developmental co-expression of duplicate gene pairs and the correlation of expression patterns with essentiality phenotype in mouse knockout experiments. When we examined the phenotypes that result from published mouse knockout experiments^29^, we found that there are duplicate gene pairs whereby each member of the pair is individually essential (lethal in a knockout experiment), duplicate pairs where one member is essential and the other is not, and duplicate pairs where neither member is essential. We hypothesised that developmental co-expression patterns are an important factor in determining the essentiality status of duplicated genes in mouse knockout experiments, since genes with overlapping developmental expression patterns are more likely to provide functional compensation for one member of a duplicate gene pair. In this study we analyse the mouse knockout data in the context of gene duplication status. We identified mouse duplicate gene pairs based on protein sequence conservation, and determined the essentiality status of these duplicated genes from published knockout experiments. Using mouse EST expression data covering 13 developmental stages, we discovered that duplicate pairs containing two essential genes tend to have greater divergence in developmental expression patterns than duplicate pairs comprised of two non-essential genes, demonstrating that developmental co-expression is an additional factor that contributes to the essentiality status of duplicated genes in the mouse.

## Results

### Datasets

We defined essential genes as those showing embryonic, perinatal, and postnatal lethality (within the first day after birth) in mouse knockout experiments. Because we wished to test the hypothesis that developmental co-expression would be a key factor to determine whether or not deletion of a duplicate gene produces an embryonic lethal phenotype, we defined essentiality based on the requirement for the gene in embryonic survival. Thus, in contrast to other studies of essential genes^22^, we did not include genes causing infertility within our essential gene dataset, but have analysed genes causing infertility as a separate group. From the knockout mouse phenotype data in the Mouse Genome Informatics (MGI) database^30^, we identified a total of 1,301 essential and 3,451 non-essential mouse genes^29^. In agreement with other reports of mouse knockout phenotypes^31,32^, our dataset shows that non-essential genes are more common than essential genes in the mouse^29^ (Table 1). In addition, within our dataset of non-essential mouse genes, a total of 1,094 genes were identified as genes that had been annotated in other studies^17,21^ as genes causing infertility (Table 1). None of the essential mouse genes in our dataset were previously annotated as an infertility gene.

**Table 1:** Numbers of mouse genes in different categories.

Genes	Total	Essential	Essential (%)	Non-essential	Non-essential (%)	Infertility	Infertility (%)
Entire Knockout dataset	4752	1301	27.38	3451	72.62	1094	23.02
Singleton	852	282	33.10	570	66.90	156	18.31
Duplicate	3900	1019	26.13	2881	73.87	938	24.05
SSD	2223	500	22.49	1723	77.51	312	14.03
WGD	1834	489	26.66	1345	73.34	549	29.93

Data presented include only the essential, non-essential and infertility genes we have identified from the literature, which have been analysed in this study. Essential (%), Non-essential (%) and Infertility (%) represent the proportions of each gene type. The infertility dataset is a sub-set of the non-essential gene dataset. The non-essential gene data columns shown here include the infertility genes.

**Figure 1:**
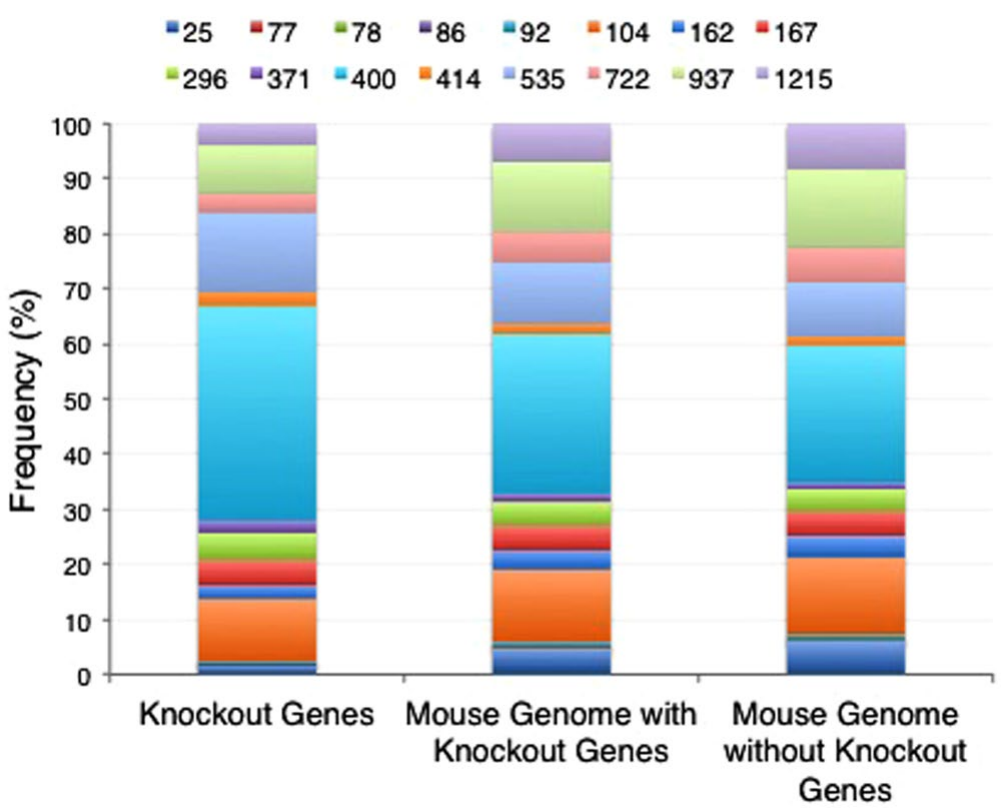
Comparison between the evolutionary age of mouse genes in our knockout dataset (all essential and non-essential genes, left column), the evolutionary age of all genes in the mouse genome including knockout genes (middle column) and the evolutionary age of all genes in the mouse genome excluding the knockout genes (right column). The numbers at the top of the chart indicate gene age in millions of years (MYr). Here, 25 MYr is the youngest taxonomic group, plotted at the bottom of each chart, and 1215 MYr is the oldest taxonomic group, plotted at the top of each chart. The ages, corresponding names of the taxonomic groups, and associated colours on the bar charts are 25= Murinae (blue), 77= Rodentia (red), 78 = Sciurognathi (green), 86 = Glires (purple), 92 = Euarchontoglires (turquoise), 104 = Eutheria (orange), 162 = Theria (dark blue), 167 = Mammalia (red), 296 = Amniota (green), 371 = Tetrapoda (purple), 400 = Euteleostomi (turquoise), 414 = Sarcopterygii (orange), 535 = Vertebrata (blue), 722 = Chordata (pink), 937 = Bilateria (green), 1215 = Opisthokonta (purple).

The evolutionary origin of gene duplicates has been proposed as a mechanism to explain the essentiality status of duplicate genes when compared to singletons^27^. Previously it was reported that the mouse knockout literature is highly enriched with evolutionarily older genes, thus underrepresenting recently duplicated genes^25^. To determine if this bias persists in the knockout dataset we utilise in this study, we compared the evolutionary ages of genes assayed in mouse knockout experiments to the ages of genes in the mouse genome as a whole (Fig. 1). The genomic evolutionary age calculations are derived from evolutionary age calculations of 11,273 mouse genes not examined in mouse knockout experiments. The ages of genes not explored in knockout experiments are presented either in combination with the mouse knockout gene dataset (middle column) or separately from the mouse knockout dataset (right column). To define the age of a gene, we used the age of the most recent duplication (MRD) event for a duplicate gene and the age of the single common ancestor (SCA) for singletons. We found that mouse knockout experiments rarely examined genes with the most ancient (Opisthokonta) or most recent (Murinae) evolutionary origins (Fig. 1, Supplementary Table 1). Within our knockout dataset, genes are heavily weighted to having evolutionary origins in the Euteleostomi, approximately 400 MYA (Supplementary Table 1). The youngest genes are also less common in the knockout set than in the whole genome. Thus, the dataset of genes examined in mouse knockout experiment does not reflect a similar balance between gene evolutionary origins as is found in the genome as a whole. As future knockout experiments are performed, the bias in evolutionary ages of genes examined in mouse knockout experiments may be diminished.

To identify duplicate gene pairs within the mouse knockout dataset, we used Ensembl gene tree analyses and pairwise BLAST searches with an E-value <10^−7^ to identify paralogous genes. From these searches we obtained 1,019 essential duplicated genes and 2,881 non-essential duplicated genes (Table 1). From the non-essential duplicated gene dataset, 938 genes were identified as duplicated genes associated with infertility. In contrast to prior reports^22,24^, we found that essential genes that are duplicates (26.13%) are less frequent than those that are singletons (33.10%) (Chi-squared p–value = 4.82 × 10^−4^). Non-essential genes are more likely to be duplicates (Table 1). It should be noted that the percentage of essential genes in this study is likely to be lower in comparison to previous reports^24,27^ because our definition of essential genes differs; we considered genes required for development as our essential gene dataset, and analysed genes associated with infertility as a separate group. We found 2,223 small–scale duplicates (essential: 500; non-essential: 1,723; Table 1) and 1,834 whole–genome duplicates (essential: 489; non-essential: 1,345; Table 1). Using mouse knockout data to define essentiality, we found 535 essential-essential (E-E), 2,489 essential-non-essential (E-NE) and 1,748 non-essential-non-essential (NE-NE) mouse duplicate gene pairs when gene duplicate pairs were defined by pairwise BLAST sequence conservation searches. Moreover, a total of 765 infertility-infertility (I-I) mouse duplicate gene pairs were found within the dataset of all NE-NE duplicate gene pairs. A total of 983 non-essential duplicate pairs with no infertility associations remained after removing the I-I pairs (NE-I).

### Differences in Developmental Expression Patterns

**Figure 2:**
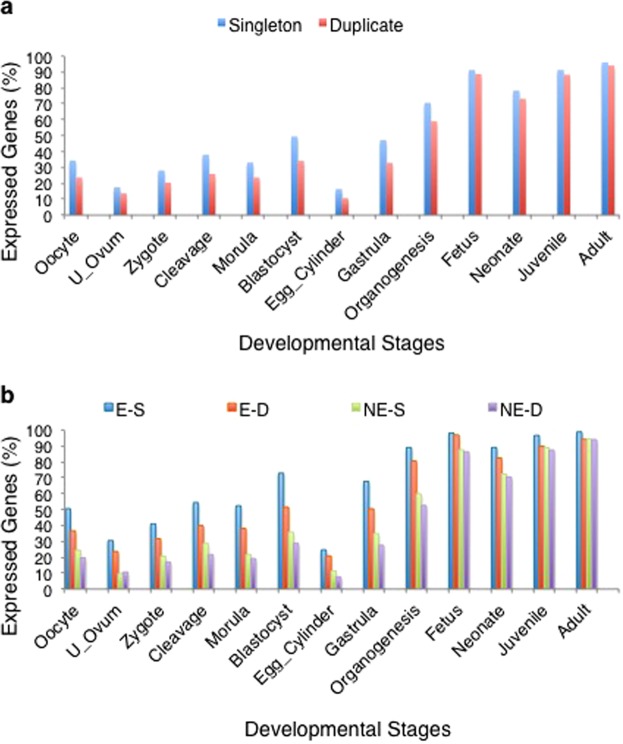
Developmental expression annotated by gene type. Frequencies (%) of all (**a**) singleton and duplicate mouse genes and (**b**) essential singleton (E-S), essential duplicate (E-D), non-essential singleton (NE-S) and non-essential duplicate (NE-D) mouse genes that are expressed across 13 stages of mouse development.

Because by definition developmental essential genes are required for an organism to survive to birth, essential genes are expected to be expressed during development. We have previously reported that a significantly greater proportion of essential genes are expressed at almost every developmental stage as compared to non-essential genes^29^. Likewise, we wanted to determine if singleton and duplicate expression patterns vary over embryonic development. Gene expression data were extracted as transcripts per million (TPM) from UniGene EST data^33^ for all essential and non-essential genes in our datasets across 13 stages of mouse development. We found that a greater proportion of genes expressed at a particular stage of development are singletons rather than duplicates (Fig. 2a; Supplementary Table 2). When segregated by both essentiality and duplication status, essential singletons are found as the most frequently expressed genes during the course of mouse development (Fig. 2b). However, a greater frequency of essential duplicates as compared to non-essential singletons are expressed at all developmental stages (Fig. 2b; Supplementary Table 3). Hence, essential genes are more likely to be expressed during development, regardless of their duplication status.

### Developmental Co–expression Analysis

To our knowledge an analysis of the essentiality status of genes within mouse duplicate gene pairs has not yet been reported. When assigning essentiality status to gene duplicates, we found that the status of members of a duplicate pair could differ, such that there were gene duplicate pairs that are both essential (producing a lethal phenotype) when knocked out individually (E-E), those that are both non-essential (producing a viable phenotype) when knocked out individually (NE-NE), and those where one member is essential and one is non-essential (E-NE). We hypothesised that overlapping developmental expression patterns (co-expression) could be an important factor in determining the essentiality of duplicate gene pairs, because genes that are expressed at different times are less likely to compensate for each other if one is knocked out. Therefore, we analysed duplicate pair developmental gene expression patterns to test our hypothesis.

**Figure 3:**
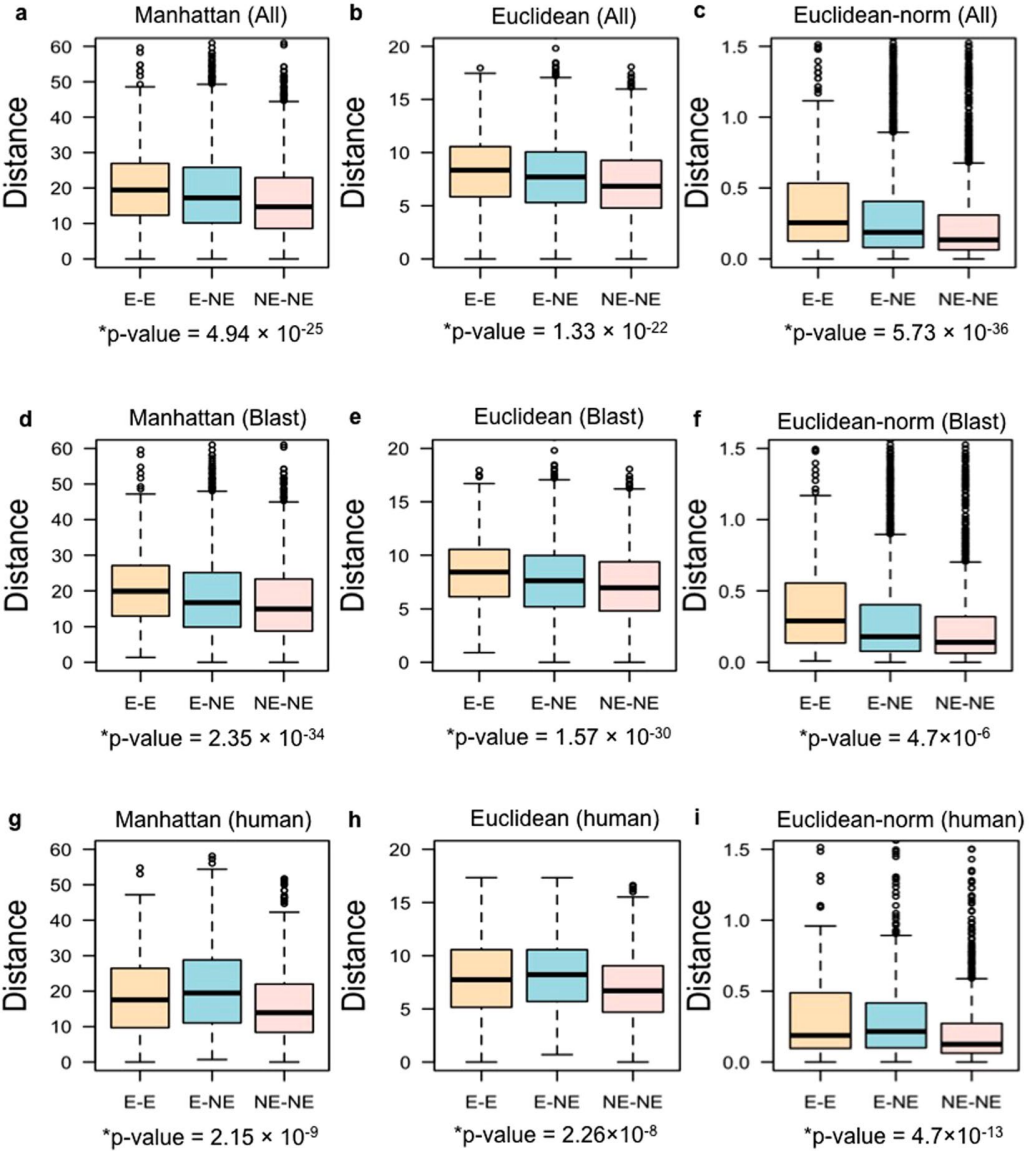
Measurements of co-expression. Differences in Manhattan (**a**,**d**,**g**) and Euclidean (**b**,**c**,**e**,**f**,**h**,**i**) distance values across 13 embryonic developmental stages between all duplicate gene pairs (**a**–**c**), all duplicate gene pairs obtained by the Blast search (**d**–**f**), and all mouse orthologues of human duplicate gene pairs (**g**–**i**) obtained from^34^. Euclidean distance was also measured using the normalised expression data (**c**,**f**,**i**). Here, distance indicates the co-expression level between duplicate gene pairs. Larger Manhattan and Euclidean distances indicate lower developmental co–expression between the two genes comprising a duplicate gene pair. The Kruskal-Wallis p-value reported below each graph shows the likelihood of E-E gene pairs to have more divergent developmental expression patterns than E-NE and NE-NE duplicate gene pairs.

We wanted to examine the developmental co-expression of duplicate gene pairs and its correlation with essentiality phenotype. Therefore, we segregated our essential and non-essential datasets into three categories of duplicate pairs based on their individual knockout phenotypes: E-E, E-NE and NE-NE duplicate gene pairs. Mouse duplicate gene pairs within our datasets were obtained from protein sequence similarity searches and also from identifying mouse orthologues of human duplicate genes pairs previously reported^34^. We computed the degree of developmental co-expression between each mouse duplicate gene and its paralogue from expression data across 13 developmental stages using the Manhattan distance method (Fig. 3a), the Euclidean distance method (Fig. 3b), or the Euclidean normalised distance (Fig. 3c). We implemented a normalisation approach for analysing Euclidean distances because in a dataset Euclidean distances tend to have many low values with a small number of high values. We therefore transformed the Euclidean distances to a log scale and normalised within the range (0, 1).

Larger Manhattan and Euclidean distances indicate lower co–expression values. We observed that E-E gene pairs tend to have higher distances and thus lower co–expression compared to NE-NE and E-NE pairs (Manhattan distance: Kruskal-Wallis p–value = 4.94 × 10^−25^; Euclidean distance: Kruskal-Wallis p–value = 1.33 × 10^−22^; normalised Euclidean distance: Kruskal-Wallis p–value = 5.73 × 10^−36^). Because we identified the genes that are members of duplicate pairs in two ways, we wished to determine if the method of duplicate identification had an effect on the outcome of our study. Co-expression of the duplicate gene pairs identified from the Blast search only (Fig. 3d–f) showed that NE–NE duplicate pairs tend to have more similar expression patterns during development, whereas E-E duplicate pairs tend to have greater divergence of expression (Manhattan distance: Kruskal-Wallis p–value = 2.35 × 10^−34^; Euclidean distance: Kruskal-Wallis p–value = 1.57 × 10^−30^; normalised Euclidean distance: Kruskal-Wallis p–value = 4.7 × 10^−6^) with E-NE pairs in between. Analysing mouse duplicate gene pairs defined by being orthologues of human duplicate gene pairs^34^ confirmed these conclusions: NE-NE duplicate gene pairs are more likely to have similar developmental co-expression patterns, as calculated using the Manhattan distance (Fig. 3g), Euclidian distance (Fig. 3h), or normalised Euclidean distance (Fig. 3i) (Manhattan distance: Kruskal-Wallis p–value = 2.15 × 10^−9^; Euclidean distance: Kruskal-Wallis p–value = 2.26 × 10^−8^; normalised Euclidean distance: Kruskal-Wallis p–value = 4.7 × 10^−13^).

**Figure 4:**
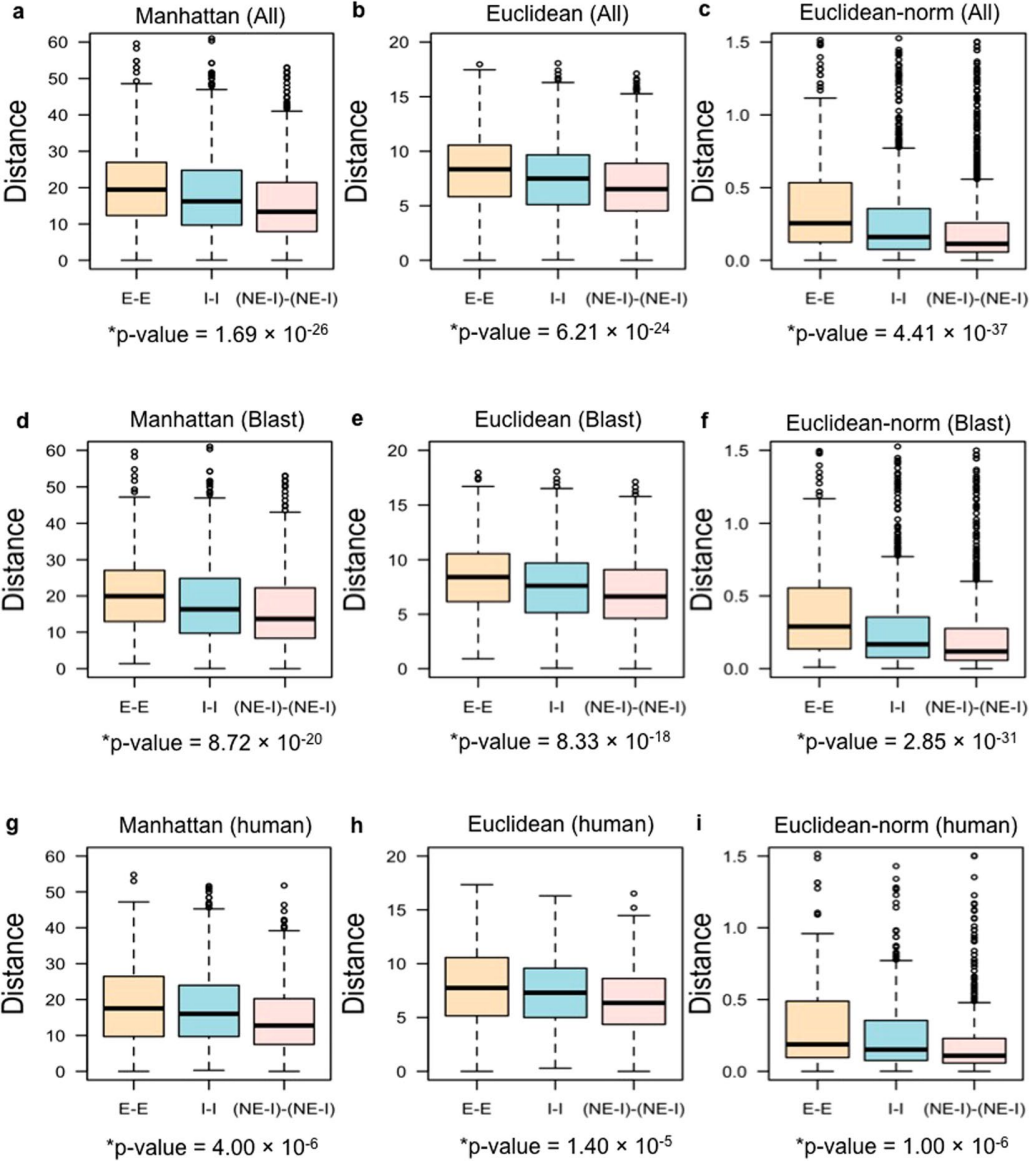
Measurements of co-expression with genes causing infertility. Differences in Manhattan (**a**,**d**,**g**) and Euclidean (**b**,**c**,**e**,**f**,**h**,**i**) distance values across 13 embryonic developmental stages between all duplicate gene pairs (**a**–**c**), all duplicate gene pairs obtained by the Blast search (**d**–**f**), and all mouse orthologues of human duplicate gene pairs (**g**–**i**) obtained from^34^. Euclidean distance was also measured using the normalised expression data (**c**,**f**,**i**). Here, distance indicates the co-expression level between duplicate gene pairs. Larger Manhattan and Euclidean distances indicate lower developmental co–expression between the two genes comprising a duplicate gene pair. (NE-I)-(NE-I) refers to all non-essential-non-essential genes pairs where no infertility-infertility (I-I) gene pair was present. The Kruskal-Wallis p-value for each analysis is reported below the corresponding graph.

Since genes associated with infertility have been considered essential from an evolutionary perspective^17,21^, we wished to determine if the inclusion or exclusion of infertility genes had an effect on the outcome of the co-expression analysis. We investigated the co-expression of I-I duplicate gene pairs in contrast to the co-expression of E-E and (NE-I)-(NE-I) duplicate pairs. (NE-I)-(NE-I) duplicate gene pairs were obtained after excluding all the I-I pairs from the total NE-NE pairs. We observed that I-I duplicate pairs tend to have higher co–expression compared to E-E gene pairs and lower co-expression than (NE-I)-(NE-I) pairs (Fig. 4a–c; Manhattan distance: Kruskal-Wallis p–value = 1.69 × 10^−26^; Euclidean distance: Kruskal-Wallis p–value = 6.21 × 10^−24^; normalised Euclidean distance: Kruskal-Wallis p–value = 4.41 × 10^−37^). Analysis of developmental co-expression of the duplicate gene pairs identified by the Blast search confirmed that co-expression values of I-I duplicate pairs tend to fall in between of E-E and (NE-I)-(NE-I) co-expression values (Fig. 4d–f; Manhattan distance: Kruskal-Wallis p–value = 8.72 × 10^−20^; Euclidean distance: Kruskal-Wallis p–value = 8.33 × 10^−18^; normalised Euclidean distance: Kruskal-Wallis p–value = 2.85 × 10^−31^). Furthermore, co-expression analysis of all mouse orthologues of human duplicate gene pairs^34^ showed that E-E duplicate pairs are more likely to have greater divergence of expression (Fig. 4g–i) compared to I-I and (NE-I)-(NE-I) duplicate pairs (Manhattan distance: Kruskal-Wallis p–value = 4.00 × 10^−6^; Euclidean distance: Kruskal-Wallis p–value = 1.40 × 10^−5^; normalised Euclidean distance: Kruskal-Wallis p–value = 1.00 × 10^−6^). Since genes associated with infertility may be required at different stages of an organism’s life (e.g. puberty) than developmental essential genes, the requirement for each paralogue in a pair of duplicated infertility genes to have overlapping developmental expression patterns may be relaxed.

**Figure 5:**
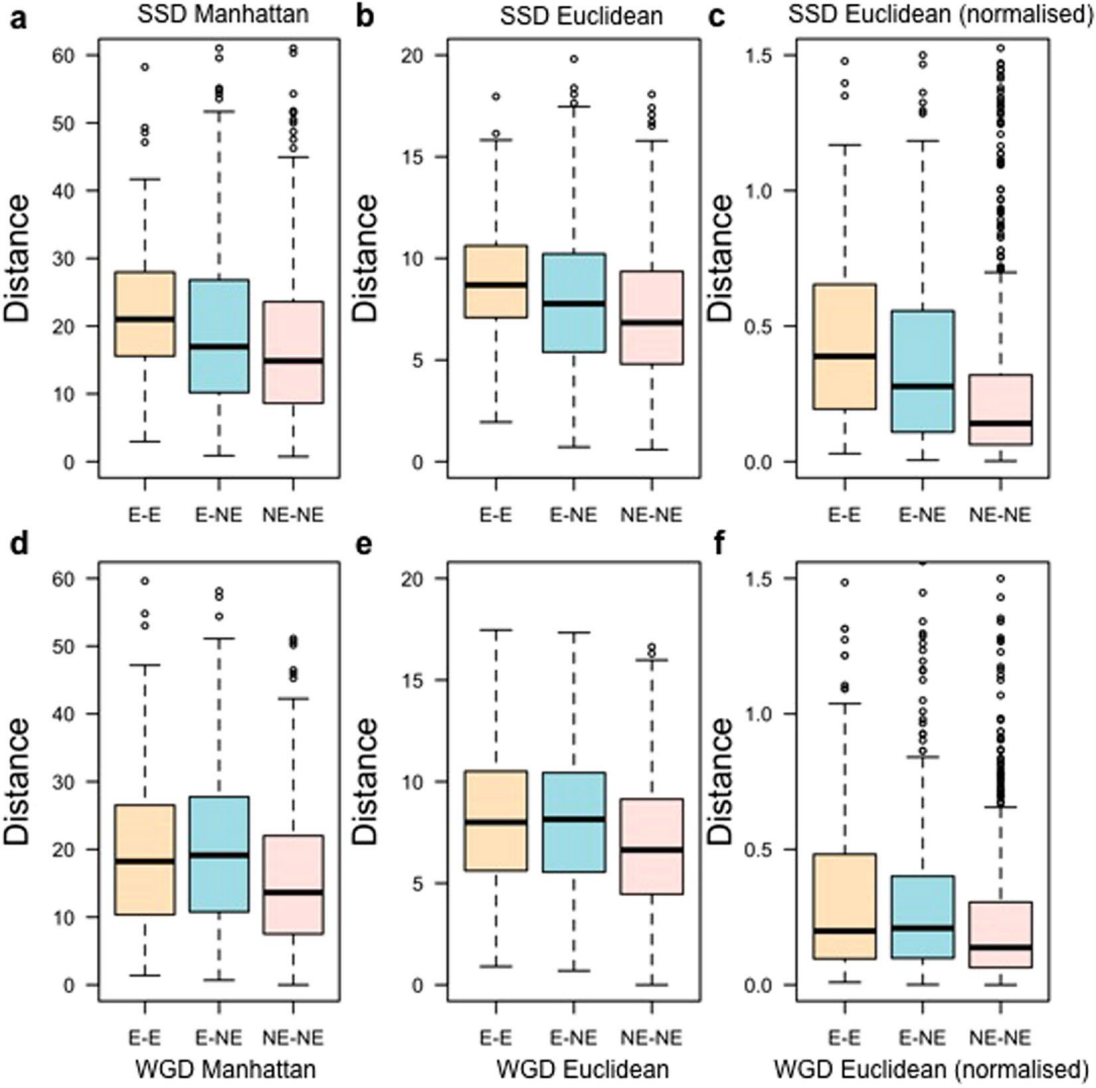
Measurements of co-expression per duplication type. Differences in the Manhattan (**a**,**d**) and Euclidean (**b**,**e**) distance values for SSD (**a**–**c**) and WGD (**d**–**f**) duplicate gene pairs across 13 embryonic developmental stages. Euclidean distance was also measured using the normalised expression data (**c**,**f**). Here, distance indicates the co-expression level between the two genes comprising a duplicate gene pair. Larger Manhattan and Euclidean distance values indicate lower co-expression profile similarities (*i*.*e* lower co-expression between genes within a duplicate gene pair).

**Figure 6:**
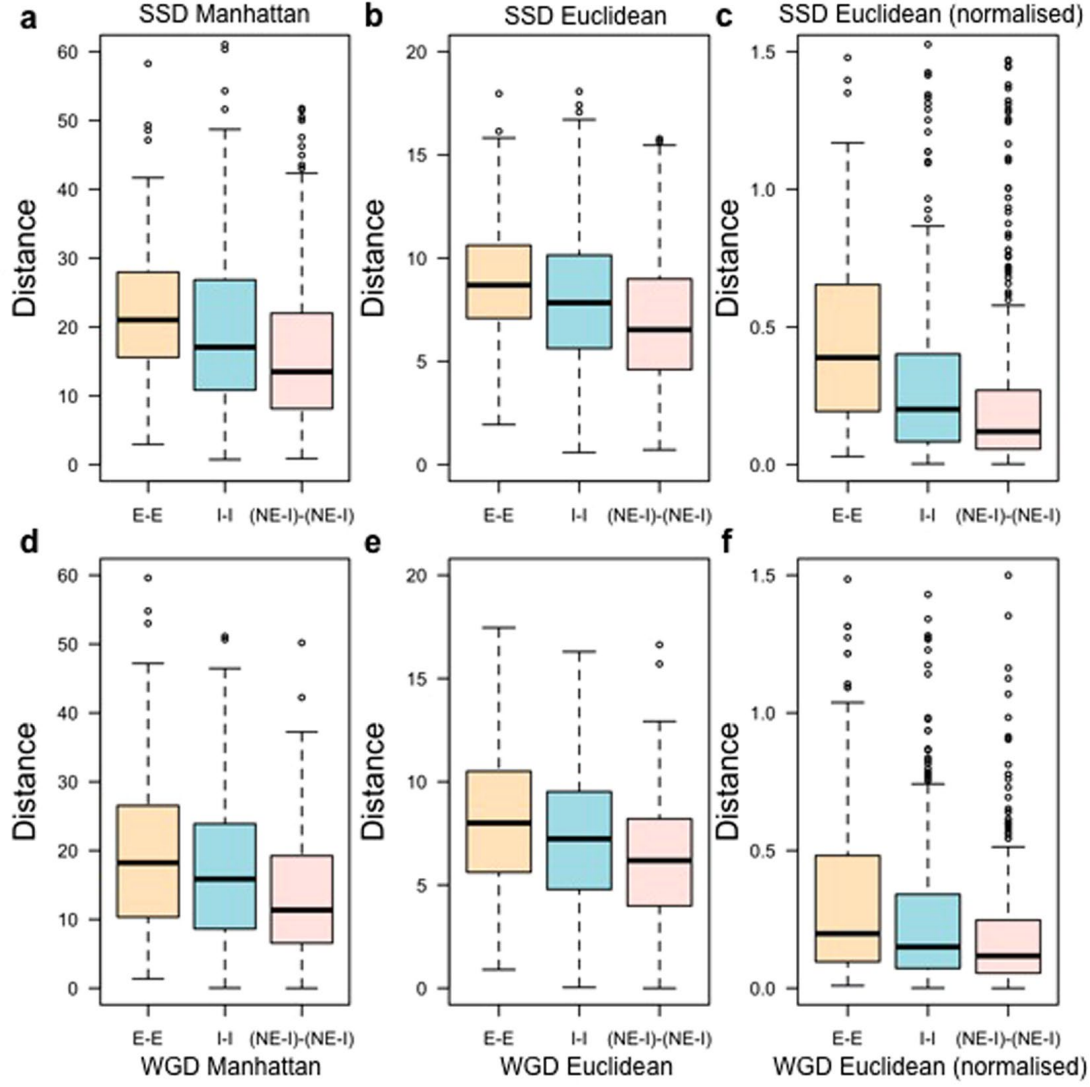
Measurements of co-expression per duplication type with genes causing infertility. Differences in the Manhattan (**a**,**d**) and Euclidean (**b**,**e**) distance values for SSD (**a**–**c**) and WGD (**d**–**f**) duplicate gene pairs across 13 embryonic developmental stages. Euclidean distance was also measured using the normalised expression data (**c**,**f**). Here, distance indicates the co-expression level between the two genes comprising a duplicate gene pair. Larger Manhattan and Euclidean distance values indicate lower co-expression profile similarities (*i*.*e* lower co-expression between genes within a duplicate gene pair).

We also ascertained that duplication type does not affect the co-expression analysis. We identified 173 E-E, 546 E-NE and 649 NE-NE mouse gene pairs duplicated by small scale duplication events (SSD). In total 194 E-E, 520 E-NE and 343 NE-NE whole genome duplication (WGD) mouse gene pairs were found within our dataset by identifying mouse duplicate gene pairs which are the orthologues of previously reported human duplicate gene pairs^34^. We found that both SSD and WGD genes followed the trend that E-E duplicate pairs tended to have more divergent developmental expression patterns than NE-NE gene pairs (Fig. 5, Supplementary Table 4). SSD gene pairs tended to have more divergence of expression than WGD gene pairs. Moreover, we identified 213 SSD and 194 WGD I-I mouse gene pairs. Analysis of the SSD and WGD duplicate pairs in terms of infertility showed that E-E gene pairs are likely to have more divergent expression patterns than (NE-I)-(NE-I) gene pairs (Fig. 6, Supplementary Table 4) with expression patterns of I-I pairs in between. Overall, these results confirm our hypothesis that duplicates with closer developmental co-expression are more likely to both be non-essential when knocked out individually. We conclude that developmental co-expression contributes to determining the essentiality status of duplicate gene pairs.

## Discussion

There has been much interest in the exploration of gene duplication and essentiality^22–25,27^. In agreement with prior studies^25,27^, we have found that mouse singleton genes are more likely to be essential than mouse duplicate genes. Although others have found that duplicate genes are as essential as singletons^22,23^, the inclusion of genes causing infertility as essential genes in their datasets may have influenced the relative proportions of essential singletons and duplicates. It was previously predicted that when the mouse knockout experimental dataset increased it would be confirmed that singletons are more likely to be essential as compared to duplicated genes^24^. The increased size of the mouse knockout experimental dataset since 2007 (approximately 1.5 times larger now than 2007) confirms this prediction, as we have identified that a greater proportion of singleton rather than duplicate genes are essential (Table 1).

Following genome duplication events, duplicate copies of genes may become redundant and lost^2^. It is postulated that for a gene to be retained after a duplication event there must be partitioning of function between the ancestral copy and duplicated copy of the gene, or the duplicate copy must acquire a new function^2,7,35^. Yet, studies have reported that duplicate copies of genes can provide functional compensation for the loss of a paralogue, and thereby both paralogues must retain similar functional capacity^36–38^. Compensation is dependent on the protein sequence conservation of paralogues, such that yeast duplicate genes with lower than 70% sequence identity behave as if they were randomly chosen pairs of singletons in genetic redundancy experiments^37^. In yeast, the protein-protein interactions that duplicate pair members participate in differ, and thus it was found that often one gene of a duplicate pair could compensate for the loss of the other, but not vice versa^38^. Likewise, in our study we found that there are duplicate gene pairs whereby one gene is essential and the other is not, suggesting that there is not absolute functional compensation of paralogous genes in the mouse.

In multi-cellular organisms, longer gestation periods and tissue complexity allows paralogues to segregate their expression patterns within embryonic tissues during development. There is evidence that developmental gene expression patterns are dynamic over the time course of development, and that genes that are expressed in early development and late development within the same organ have different functional annotations^39^, thereby allowing appropriate specialisation of developing tissues. We hypothesised that compensation or buffering between mouse duplicate genes would depend upon the occurrence of developmental co-expression between duplicate partners. Indeed, gene expression levels have been identified as strong predictors of buffering^36^. Consistent with our hypothesis, we found that duplicate gene pairs where each member is essential when knocked out individually display low levels of developmental co-expression. A limitation of our study is the lack of tissue-specific and cell-type specific gene expression datasets over multiple stages of mouse development, such that the co-expression specificity we have analysed is limited to developmental stage. Even with this caveat, we find that developmental co-expression is strongly correlated with the essentiality status of mouse duplicate gene pairs. Developmental co-expression is thus identified as a new factor that explains the essentiality status of mouse duplicate pairs. We propose that a lack of developmental co-expression prevents buffering between these duplicates, rendering each gene essential. These analyses provide additional insights into the relationship between gene duplication and essentiality, providing an explanation for the diversity of experimental outcomes in mouse knockout experiments with members of a duplicate gene pair.

## Methods

### Essential and Non-essential Mouse Gene Datasets

We defined essential genes (E) as those that cause lethality prior to postnatal day 1 in a single gene knockout (targeted deletion) experiment. We used the prenatal (MP:0002080), perinatal (MP:0002081), and postnatal (MP:0002082) lethal mouse knockout phenotype annotations from the MGI database^30^ to mark a mouse gene as essential. We considered 18 different viable mouse knockout phenotypes to define non-essential mouse genes (NE) (Supplementary Table 5). Additional details of our essential and non-essential datasets have been previously reported^29^. In contrast to other studies^23,27^, we did not classify genes causing infertility to be essential genes, but analysed infertility genes as a separate dataset. Infertility genes (I) were obtained from published reports^23,27^. This list of infertility causing genes was compared with our essential and non-essential genes lists. Genes within our NE dataset that were annotated as I in other publications were removed from the NE dataset for analyses (Supplementary Table 6). The remaining non-essential genes were treated as a separate group (NE-I). None of our essential genes were annotated in other publications as infertility genes.

### Singletons and Duplicates

We used Ensembl (release 75) gene trees of mouse gene families to categorise and label all mouse protein-coding genes into two groups: singletons and duplicates. Essentiality status was assigned to these singleton and duplicate genes by analysing their knockout phenotype annotations from the MGI database. Genes without confirmed essentiality status from MGI were removed from the dataset for subsequent analysis. This resulted in four datasets: (i) essential singletons, (ii) non-essential singletons, (iii) essential duplicates, and iv) non-essential duplicates. The paralogues of the mouse genes present in the mouse essential and non-essential duplicate datasets were then identified, by measuring protein sequence similarity based on BLAST search. We downloaded the BLAST+ software package from the NCBI database and performed Blast search^40^ on our local computer to detect mouse duplicate gene pairs within our own duplicate datasets. We identified three categories of duplicate gene pairs: (i) essential-essential (E-E), (ii) non-essential-non-essential (NE-NE), and (iii) essential-non-Essential (E-NE). Two all-against-all BLAST searches were performed on the essential duplicate and non-essential duplicate datasets to generate the E-E and NE-NE gene pairs respectively. The genes in the essential duplicate dataset were then searched against the database of the non-essential duplicate dataset using BLAST to generate the E-NE gene pairs. In accordance with prior studies^27,34^, we identified duplicate partners of a mouse gene within our datasets by considering sequence similarity scores and E-values (<10^−7^) from the BLAST search. The best match from the BLAST output was then considered to be the closest paralogue for a mouse duplicate gene. In addition, we used human duplicate gene pairs listed in Makino and MyLysaght^34^ as another means to define parologous genes. Mouse orthologues of these human duplicate genes were obtained from the Ensembl BioMart data-mining tool^41^ (http://www.ensembl.org/biomart/martview/) with the Ensembl release 75 dataset of the *Homo sapiens* genes (GRCh37.p13). We included only one-to-one mouse orthologues of these human duplicate genes.

A mouse duplicate gene was further classified as either a small–scale duplicate (SSD) or a whole–genome duplicate (WGD). A gene was defined as a WGD if its human orthologue was found within the 9,059 human WGD duplicate pairs listed in Makino and MyLysaght^34^. The rest of the mouse duplicate genes in our datasets were classified as small–scale duplicates.

### Total Mouse Gene Dataset

All genes in the mouse genome were obtained from the Mouse Genome Informatics (MGI) database^30^ (http://www.informatics.jax.org/phenotypes.shtml). All essential and non-essential genes that we examined^29^ in this study were also included in the whole mouse gene dataset. Mouse genes that were not classified as either essential or non-essential were classified as genes with unknown essentiality status.

### Evolutionary Age

Evolutionary ages of mouse protein coding genes were retrieved from Ensembl (release 75) gene trees, which represent the evolutionary processes by which genes diverged from their common ancestors, as previously described^29^. We assigned two evolutionary ages to each duplicated gene in our datasets: the age of the most recent duplication (MRD) event and the age of the evolutionarily most distantly related species, *i*.*e*., the age of the duplicate common ancestor (DCA) that has an identified orthologue of that gene. For singletons, we used the age of their single common ancestor (SCA). Evolutionary ages are reported in millions of years (MYA).

### Gene Expression During Development

Using a methodology we have previously described^29^, raw expression data of mouse essential and viable genes were obtained directly from the NCBI UniGene database^33^ as expressed sequence tag (EST) clusters using UniGene IDs. We extracted EST clusters from 13 developmental stages: oocyte, unfertilized ovum, zygote, cleavage, morula, blastocyst, egg cylinder, gastrula, organogenesis, fetus, neonate, juvenile and adult. Since the total number of ESTs for a particular gene varies greatly between different developmental stages, the raw data in UniGene have been normalised to get gene expression in the form of transcripts per million (TPM), and we utilised the TPM values in this study. Every gene with a minimum Transcript per Million (TPM) value of 1 was defined as expressed at a particular stage. Eq. 1 was used to calculate a TPM for the *i*^*th*^ gene at *j*^*th*^ developmental stage^33^.

1 $$TP{M}_{i}^{j}=\frac{Number\,of\,ESTs\,for\,{i}^{th}\,gene\,in\,{j}^{th}\,stage}{Total\,ESTs\,in\,{j}^{th}\,stage}\times {10}^{6}$$

TPMs were also transformed to their corresponding log values using Eq. 2 to measure co-expressions between every gene pair.

2 $${L}_{TP{M}_{i}^{j}}=lo{g}_{e}\,(TP{M}_{i}^{j}+1)$$

TPMs were also normalised within the range (0, 1) using Eq. 3, dividing each TPM by the maximum TPM value.

3 $${N}_{TP{M}_{i}^{j}}=TP{M}_{i}^{j}/max(TPM)$$

We used the Euclidean and the Manhattan distance methods to calculate numerical scores representing gene co-expression. These numerical distance values are used to compare gene expression between every gene pair during development. If ${\boldsymbol{a}}=({a}_{1},\,{a}_{2}\ldots .\,{a}_{13})\,\,$ and ${\boldsymbol{b}}=({b}_{1},{b}_{2}\ldots .{b}_{13})$ are two mouse genes with expression values across 13 developmental stages, then the Euclidean and Manhattan distances between them were calculated by Eqs 4 and 5, respectively. Small scores (distances) reflect higher co-expression between genes. We utilised both log (Eq. 2) and normalised TPM data (Eq. 3) to compute the Euclidean distance to evaluate the possibility that the scale of data would affect the conclusion drawn from the Euclidean distance analysis.

4 $$EucDis({\boldsymbol{a}},{\boldsymbol{b}})=\sqrt{\sum _{i=1}^{13}{({a}_{i}-{b}_{i})}^{2}}$$

5 $$ManDis({\boldsymbol{a}},{\boldsymbol{b}})=\sum _{i=1}^{13}|{a}_{i}-{b}_{i}|$$

### Statistical Tests

The nonparametric Kruskal-Wallis^42^ method was used to test the co-expression patterns of duplicate gene pairs. This statistical test was used because our co-expression datasets did not show a normal distribution. This statistical test was carried out using the statistical software package SPSS^43^ version 22. The Chi–square (*χ*^2^) test was carried out to investigate whether singleton and duplicate genes are differentially expressed across different developmental stages. The null hypothesis tested by the Chi-square test was “the frequencies of singleton and duplicate genes expressed at one developmental stage are equal.” To calculate expected values for each developmental stage, the total number of genes expressed at that stage (total of singletons and duplicates) was divided by the total of all genes in our dataset and the relative proportion of singleton and duplicate genes expressed at that particular stage was calculated. The total number of singletons in our dataset was then multiplied by this gene proportion value to determine the expected singleton frequency at that stage. The same procedure was used to calculate the expected frequency of duplicate genes. Thus by simple proportions from the totals we found an expected number to compare each observed number. We then used the observed and expected frequencies to calculate the Chi-square test statistic. The Bonferroni correction^44^ was applied to calculate corrected p–values.

## Supplementary information

Supplementary Information

Supplementary Dataset

## Data Availability

All data generated or analysed during this study are included in this published article (and its Supplementary Information files).
