# Supplementary material for: The Essentiality Status of Mouse Duplicate Gene Pairs Correlates with Developmental Co-Expression Patterns
Source: Sci Rep. 2019 Mar 1;9:3224. doi: 10.1038/s41598-019-39894-9 (PMC6397145; doi:10.1038/s41598-019-39894-9)
Supplement: Supplementary file 1 — Supplementary Information [file 41598_2019_39894_MOESM1_ESM.docx]

**The Essentiality Status of Mouse Duplicate Gene Pairs Correlates with Developmental Co-Expression Patterns**

**Mitra Kabir^1^, Stephanie Wenlock^1,2,^, Andrew J. Doig^3^, Kathryn E. Hentges^1*^**

1 – Division of Evolution and Genomic Sciences, Faculty of Biology, Medicine and Health, Manchester Academic Health Science Centre, University of Manchester, Oxford Road, Manchester, M13 9PT, UK

2 – Present address: Department of Pathology, Cambridge Genomic Services, University of Cambridge, Cambridge, CB2 1TN, UK

3 – Manchester Institute of Biotechnology and School of Biological Sciences, Faculty of Biology, Medicine and Health, Manchester Academic Health Science Centre, University of Manchester, 131 Princess Street, Manchester, M1 7DN, UK

* = Correspondence to Kathryn.Hentges@manchester.ac.uk

Supplementary Table 1: Chi-square statistical test results presenting frequencies of knockout genes versus all genes in the mouse genome for different evolutionary age groups. The p-value for the Bonferroni correction is 0.003125. Here, K refers to the knockout genes in our dataset (all essential and non-essential genes), MK refers to all genes all genes in the mouse genome including knockout genes and MwK refers to all genes in the mouse genome excluding the knockout genes.

| **Taxon** | **MRD age** | **K (%)** | **MK (%)** | **MwK (%)** | **p-value (K vs MK)** | **p-value (K vs MwK)** |
| --- | --- | --- | --- | --- | --- | --- |
| Murinae | 25 | 1.75 | 4.77 | 6.01 | 3.3×10^-19^ | 4.4×10^-29^ |
| Rodentia | 77 | 0.06 | 0.02 | 0.00 | 0.11 | 6.9×10^-3^ |
| Sciurognathi | 78 | 0.04 | 0.21 | \| 0.27 \| \| --- \| | 1.7×10^-2^ | 3.5×10^-3^ |
| Glires | 86 | 0.11 | 0.21 | 0.25 | 0.17 | 7.7×10^-2^ |
| Euarchontoglires | 92 | 0.52 | 0.70 | 0.78 | 0.17 | 7.3×10^-2^ |
| Eutheria | 104 | 11.33 | 13.20 | 13.97 | 1.7×10^-3^ | 3.1×10^-5^ |
| Theria | 162 | 2.37 | 3.48 | 3.93 | 2.4×10^-4^ | 1.7×10^-6^ |
| Mammalia | 167 | 4.60 | 4.46 | 4.40 | 0.69 | 0.59 |
| Amniota | 296 | 4.96 | 4.32 | 4.05 | 6.8×10^-2^ | \| 1.2×10^-2^ \| \| --- \| |
| Tetrapoda | 371 | 2.24 | 1.50 | \| 1.19 \| \| --- \| | 5.1×10^-4^ | 7.6×10^-7^ |
| Euteleostomi | 400 | 38.91 | 28.97 | 24.89 | 1.7×10^-26^ | 2.4×10^-50^ |
| Sarcopterygii | 414 | 2.48 | 1.82 | 1.54 | 4.5×10^-3^ | 6.6×10^-5^ |
| Vertebrata | 535 | 14.44 | 11.16 | 9.81 | 1.2×10^-8^ | 2.1×10^-15^ |
| Chordata | 722 | 3.45 | 5.53 | 6.38 | 3.4×10^-8^ | 9.9×10^-13^ |
| Bilateria | 937 | 8.72 | 12.62 | 14.22 | 9.3×10^-12^ | 6.9×10^-19^ |
| Opisthokonta | 1215 | 4.01 | 7.05 | 8.29 | 5.9×10^-13^ | 2.5×10^-20^ |

Supplementary Table 2. Chi-square statistical test results presenting frequencies of singleton versus duplicate mouse genes expressed at different developmental stages**.** The Chi-square p-value for the Bonferroni correction is 0.00385. For each observed gene frequency (either singleton or duplicate) at one particular stage, the respective expected frequency was calculated by multiplying the proportion of genes expressed at that particular stage by the total of that gene type in our dataset.

| **Developmental stage** | **Singleton (%)** | **Duplicate (%)** | **p-value** |
| --- | --- | --- | --- |
| **Oocyte** | 34.3 | 24.6 | 1.5×10^-10^ |
| **Unfertilized Ovum** | 17.5 | 13.7 | 2.3×10^-3^ |
| **Zygote** | 28.2 | 20.5 | 6.4×10^-7^ |
| **Cleavage** | 38.1 | 25.9 | 6.1×10^-12^ |
| **Morula** | 33.1 | 23.6 | 1.3×10^-8^ |
| **Blastocyst** | 49.8 | 34.1 | 2.4×10^-14^ |
| **Egg Cylinder** | 16.4 | 10.7 | 3.5×10^-7^ |
| **Gastrula** | 47.2 | 32.9 | 2.7×10^-13^ |
| **Organogenesis** | 70.6 | 59.0 | 5.1×10^-6^ |
| **Fetus** | 91.2 | 88.7 | 0.41 |
| **Neonate** | 78.3 | 73.2 | 0.07 |
| **Juvenile** | 91.3 | 88.2 | 0.31 |
| **Adult** | 95.9 | 93.9 | 0.54 |

Supplementary Table 3: Chi-square statistical test results presenting differences in proportions of essential singleton versus essential duplicate and non-essential singleton versus non-essential duplicate mouse genes. The Chi-square p–value for the Bonferroni correction is 0.00385. At one particular developmental stage, the expected frequency of a gene type (either singleton or duplicate) was calculated by multiplying the proportion of genes expressed at that particular stage by the total of that gene in our dataset.

| **Developmental stage** | **Essential** | | | **Non-essential** | | |
| --- | --- | --- | --- | --- | --- | --- |
|  | **Singleton**  **(%)** | **Duplicate**  **(%)** | **p-value** | **Singleton**  **(%)** | **Duplicate**  **(%)** | **p-value** |
| **Oocyte** | 50.60 | 36.63 | 1.4×10^-4^ | 24.58 | 19.66 | 8.0×10^-3^ |
| **Unfertilized Ovum** | 30.48 | 23.63 | 0.02 | 9.88 | 10.82 | 0.48 |
| **Zygote** | 41.04 | 31.75 | 7.0×10^-3^ | 20.72 | 17.06 | 0.04 |
| **Cleavage** | 54.38 | 39.88 | 1.8×10^-4^ | 28.67 | 21.75 | 3.7×10^-4^ |
| **Morula** | 52.39 | 38.13 | 1.3×10^-4^ | 21.81 | 19.19 | 0.15 |
| **Blastocyst** | 72.91 | 51.50 | 1.0×10^-6^ | 35.90 | 28.77 | 1.4×10^-3^ |
| **Egg Cylinder** | 24.70 | 20.88 | 0.17 | 11.45 | 7.60 | 9.4×10^-4^ |
| **Gastrula** | 67.73 | 50.50 | 5.8×10^-5^ | 34.94 | 27.61 | 6.7×10^-4^ |
| **Organogenesis** | 89.04 | 80.50 | 0.10 | 59.88 | 52.58 | 0.01 |
| **Fetus** | 98.01 | 96.88 | 0.84 | 87.59 | 86.35 | 0.76 |
| **Neonate** | 89.04 | 82.38 | 0.21 | 72.29 | 70.53 | 0.59 |
| **Juvenile** | 96.61 | 89.75 | 0.20 | 88.92 | 87.44 | 0.69 |
| **Adult** | 98.90 | 94.13 | 0.40 | 94.22 | 93.99 | 0.96 |

Supplementary Table 4: Kruskal-Wallis statistical test results of the SSD and WGD co–expression analyses. Here, SSD(I) and WGD(I) refer to the analyses in which co-expression of the infertility-infertility (I-I) mouse duplicate gene pairs were investigated.

| **Duplicate gene pair** | **p-value** | | |
| --- | --- | --- | --- |
|  | **Manhattan distance** | **Euclidean distance** | **Euclidean distance**  **(normalised)** |
| SSD | 5.2×10^-9^ | 5.4×10^-8^ | 1.9×10^-24^ |
| WGD | 1.5×10^-5^ | 4.7×10^-5^ | 4.3×10^-8^ |
| SSD(I) | 6.7×10^-10^ | 1.2×10^-8^ | 1.2×10^-21^ |
| WGD(I) | 6.9×10^-7^ | 2.0×10^-6^ | 4.0×10^-6^ |

**Supplementary Table 5:** Mammalian Phenotype (MP) annotations from the MGI database that were used for defining genes as non-essential.

| **Gene Type** | **MP Term** | **MP ID** |
| --- | --- | --- |
| Non-essential | pigmentation phenotype | MP:0001186 |
| Non-essential | abnormal immune system physiology | MP:0001790 |
| Non-essential | abnormal touch/nociception | MP:0001968 |
| Non-essential | abnormal skin morphology | MP:0002060 |
| Non-essential | abnormal postnatal growth/weight/body size | MP:0002089 |
| Non-essential | altered tumor susceptibility | MP:0002166 |
| Non-essential | normal phenotype | MP:0002873 |
| Non-essential | premature aging | MP:0003786 |
| Non-essential | abnormal behaviour | MP:0004924 |
| Non-essential | abnormal eye physiology | MP:0005253 |
| Non-essential | adipose tissue phenotype | MP:0005375 |
| Non-essential | homeostasis/metabolism phenotype | MP:0005376 |
| Non-essential | hearing/vestibular/ear phenotype | MP:0005377 |
| Non-essential | behaviour/neurological phenotype | MP:0005386 |
| Non-essential | taste/olfaction phenotype | MP:0005394 |

**Supplementary Table 6: Lists of mouse essential, non-essential and infertility genes (see separate excel file).**
